# Supplementary material for: A dual-branch model combining convolution and vision transformer for crop disease classification
Source: PLoS One. 2025 Apr 24;20(4):e0321753. doi: 10.1371/journal.pone.0321753 (PMC12021192; doi:10.1371/journal.pone.0321753)
Supplement: Attachment — Submitted filename: Response to Reviewers .docx [file pone.0321753.s002.docx]

Dear editor and reviewers:

Thank you for offering us an opportunity to improve the quality of our submitted manuscript “A dual-branch model combining convolution and Vision Transformer for crop disease classification”. We appreciate very much the editor's and reviewers’ constructive and insightful comments. In this revision, we have addressed all of these comments. We hope the revised manuscript has now met the publication standard of your journal.

Revised portion are marked in red in the paper. The main corrections in the paper and the responses to the editor's and reviewers’ comments are as following:

**Editor**

Thank you for submitting your manuscript to PLOS ONE. After careful consideration, we feel that it has merit but does not fully meet PLOS ONE’s publication criteria as it currently stands. Therefore, we invite you to submit a revised version of the manuscript that addresses the points raised during the review process. Also address the following comments while revising your paper:

**1. Comment**: Briefly mention how the model outperforms state-of-the-art approaches, emphasizing its advantages in accuracy and computational efficiency.

**Reply**: Thank you for your advice. We have highlighted the advantages of model performance as well as computational efficiency in the abstract, results discussion section, and conclusion.

**2. Comment**: Clarify the technical importance of integrating the ALP-FFN module and self-attention mechanism for improved performance.

**Reply**: We have supplemented the description of the advantages of linear self-attention and ALP‑FFN modules in section 2.3.2 and section 2.3.3, and conducted additional ablation experiments to verify the effectiveness of the two modules. As a result, the original section 3.3 is now divided into two parts: section 3.3.1 “effectiveness of the dual-branch architecture” and section 3.3.2 “effectiveness of the ALP-FFN module and the SSA module respectively”. Furthermore, we have removed the description of the ALP-FFN module’s parameter reduction from both the introduction and section 2.3.3, since newly added ablation experiments indicate that the deep convolution operation introduced by ALP-FFN slightly increases the number of parameters. We apologize for this oversight.

**3. Comment**: Provide context for the reported accuracy and efficiency, explaining how these advancements address specific challenges in crop disease identification or precision agriculture.

**Reply**: Thank you for your suggestion. In the introduction, we have addressed the current challenges in disease classification, including the complexity of crop disease characteristics, the difficulty of integrating CNNs with ViTs, and the unsuitability of ViTs for resource-constrained devices. We also explained in the methods and results sections why our model can effectively tackle these challenges.

**Reviewer 1**

**1. Comment**: The most important pre-processing techniques for agricultural images are not shown.

**Reply**: We have changed the title of section 2.1 from “Datasets” to “Datasets and preprocessing”, and expanded this section to include explanations of image preprocessing techniques, encompassing dataset partitioning, image cropping, image normalization. In addition, we have described the data augmentation techniques used in the training process in section 3.1.

**2. Comment**: Add additional research to review the previous research review for the research field.

**Reply**: We have reviewed applied research on combining CNNs with transformers for crop disease identification and reviewed their shortcomings. Also, we added references to the following literature:

Reference 24: Xie W, Zhao M, Liu Y, Yang D, Huang K, Fan C, et al. Recent advances in Transformer technology for agriculture: A comprehensive survey. Eng Appl Artif Intell. 2024;138: 109412. doi:10.1016/j.engappai.2024.109412.

Reference 26: Yu S, Xie L, Huang Q. Inception convolutional vision transformers for plant disease identi-fication. Internet Things. 2023;21: 100650. doi:10.1016/j.iot.2022.100650.

Reference 27: Wang Y, Chen Y, Wang D. Convolution Network Enlightened Transformer for Regional Crop Disease Classification. Electronics. 2022;11: 3174. doi:10.3390/electronics11193174.

**3. Comment**: Please explain figure 7 better.

**Reply**: We apologize for any confusion caused by Figure 7. We have explained the calculation process of the ALP-FFN module in more detail and added the calculation formulas. In addition, we noticed that figure 7 is poorly drawn, so we have redrawn figure 7 to make it more understandable.

**4. Comment**: Discuss the results listed in the tables and the criteria that were adopted to establish a clear vision.

**Reply**: We have improved the discussion of the experimental results in sections 3.2.1 and 3.2.2, and added new discussion in comparison with previous work. In addition, the explanation of the performance metrics has been given in section 3.2.

**5. Comment**: Clarification of proposed future work from this manuscript.

**Reply**: In the conclusion section, we have outlined our future work plan as follows:

(1) Collecting datasets from real field environments.
(2) Expanding the model to include more crop and disease types.

**Reviewer 2**

**1. Comment**: Your figures appears as an appendix, fix them within the papers even if it takes a whole page.

**Reply**: Thank you for your advice. However, the journal requires that figures cannot be included in the main manuscript file. Each figure must be prepared and submitted as a separate document.

**2. Comment**: Your tables and format is not well arranged, make your tables in a page, not in separate pages.

**Reply**: We have redesigned and adjusted the format and layout of all the tables to ensure that each table is fully displayed on its own page.

**3. Comment**: Follow the actual format.

**Reply**: We have carefully revised the manuscript format to meet the journal requirements.

Thank you again for your positive and constructive comments and suggestions on our manuscript.

We hope you will find our revised manuscript acceptable for publication.
